# Perceived social support and compliance on stay-at-home order during COVID-19 emergency in Nepal: an evidence from web-based cross-sectional study

**DOI:** 10.1186/s12889-023-15396-2

**Published:** 2023-03-21

**Authors:** Namuna Shrestha, Reena Koju, Dirghayu K.C., Namra Kumar Mahato, Anil Poudyal, Ranjeeta Subedi, Nitisha Gautam, Anju Vaidya, Shristi Karki

**Affiliations:** 1 Public Health Promotion and Development Organization, Kathmandu, Nepal; 2 Public Health and Environment Research Center, Kathmandu, Nepal

**Keywords:** COVID-19 pandemic, Nepal, Perceived support, Stay-at-home order

## Abstract

**Background:**

After COVID-19 was declared a Public Health Emergency of International Concern by WHO, several non-pharmaceutical interventions were adopted for containing the virus. Success to which largely depend upon citizens’ compliance to these measures. There is growing body of evidence linking social support with health promoting behaviour. Hence, this research aimed to study the effects on compliance with stay-at-home order in relation to their perceived social support.

**Methods:**

A web-based cross-sectional study was conducted among adult participants aged 18 years and above residing in Bagmati Province, Nepal. A convenient non-probability sampling method was adopted to select the required number of samples. The questionnaire was developed through an extensive review of literature, and consultations with the research advisor, subject experts, as well as peers and converted to online survey form using Google Forms. Perceived social support was measured using the Multidimensional Scale of Perceived Social Support (MSPSS) scale whereas compliance was assessed using a single screening question. Statistical analysis was performed using SPSS version 20 involving both the descriptive and inferential statistics.

**Results:**

Two fifth (40.2%) of the participants reported poor compliance with stay-at-home order which was found higher among participants who were not vaccinated against COVID-19 compared to those vaccinated (p value < 0.05). A significant difference was observed between sex and perceived support (p value < 0.05) with higher proportion (80.8%) of female participants reporting perceived support from family, friends, and significant others in comparison to male participants.

**Conclusion:**

Overall, the results of this study suggest that the perceived support from family is higher compared to others. Further evidence might be helpful to understand contextual factors on compliance with public health measures. Tailoring behaviour change messages as per the community needs would help the response in such emergencies. The findings from this study might be useful as one of the evidence base for formulating plans and policy during emergencies of similar nature.

**Supplementary Information:**

The online version contains supplementary material available at 10.1186/s12889-023-15396-2.

## Introduction

The novel coronavirus also referred to as severe acute respiratory syndrome coronavirus-2 (SARS-CoV-2) caused a severe respiratory disease known as coronavirus disease (COVID-19), which started as an outbreak in Wuhan of Hubei province of China. By 30 January 2019, WHO declared the outbreak a Public Health Emergency of International Concern [1]. As of August 2020, the novel coronavirus 2019 (COVID-19) had spread to every region of the world except Antarctica infecting millions of people and killing thousands. Nepal’s first COVID-19 case was confirmed on January 23, and the country continued to see a gradual increase in the number of confirmed cases. As of 31st January 2022, Nepal had 950,441 confirmed cases of COVID-19 with 854,707 recovered cases and 11,735 deaths among the total cases [2].

While no treatment has yet been identified, prevention is crucial to minimize the risk posed. The World Health Organization (WHO), national and international health agencies, and other experts have published guidance on best practices. Several non-pharmaceutical interventions such as isolation, contact tracing, quarantine, and social (physical) distancing have been shown to be effective in containing the virus [3, 4]. In the context of developing countries, adopting targeted social distancing measures combined with testing has proven to be a more effective strategy [5]. To gain control over this pandemic scenario, many public health measures have been initiated at the national, provincial, and local levels to combat COVID-19 disease in Nepal [6]. Adopting the global trend for the efficient containment of COVID-19, the government of Nepal responded to the pandemic through the implementation of large-scale social restrictions under the Nepal Public Health Service Act 2075 (Clause 48.4) which include the non-pharmaceutical interventions, including border control, lockdown, social distancing, and stay at home, helped Nepal in preventing the spread of COVID-19 [7]. The Government of Nepal in almost all provinces and territories, imposed restrictions aimed at promoting social distancing including a ban on large gatherings including religious, cultural as well as travel, and related activities. Non-adherence to social distance, physical distance, and self-isolation during an epidemic are examples of social behaviors that undoubtedly would have decelerated containment of COVID-19 super-spreading scenarios [8].

People’s adherence to control measures is affected by their knowledge, attitudes, and practices toward COVID-19 [9]. Citizens’ perceptions of the government’s response to the COVID-19 situation and subsequent recovery efforts are important in the context of the implicit or explicit response to the social distancing policy. Adherence results may depend on a number of factors. In the context of personal health compliance behavior, individuals follow health care recommendations based on their perception of the severity and impact of a situation [10, 11]. Based on the health policy recommendation, individuals will evaluate the effectiveness of the recommendations and whether they have the capabilities to manage the process. Since the COVID-19 environment is the worst of what the world has experienced to date, adaptive action depends on the cognitive assessment processes of the situation and the different sensitivities, vulnerabilities, interpretations, as well as the reactions to the situation [12].

The COVID-19 situation is not limited to individuals only; it extends to the social fabric. Policy recommendations have social implications because restricting contact can disrupt social group activities such as parties, gatherings, and shopping at malls. Therefore, personal behavior is affected by social norms and practices.

Evidence worldwide suggests the role of social support in adopting health promoting behaviours [13–15]. In addition, increase in social support has been linked with reduction in stress and thereby promoting their well-being and quality of life [16]. Considering the available literature that exemplifies the role of social support for health behavior change [17, 18], the current study aimed to assess the level of compliance with perceived social support and home stay advice among residents of Nepal.

## Methodology

### Sample size determination and sampling procedure

A cross-sectional descriptive study using quantitative methods was conducted among participants aged 18 years and above residing in Bagmati province, Nepal. This province was chosen as a study site because of its high population density and the number of cases of COVID-19 (at the time of conducting this study) in this province compared to other provinces. A sample size of 215 was estimated including an adjustment for 5% non-response to the initial estimate of 204. A convenient non-probability sampling method was adopted to select the required number of samples.

### Data collection

An online survey form was prepared using Google Forms. The questionnaire was developed based on an extensive review of literature, consultations with the research advisor, subject expert, research expert, and peers. Research experts consulted included professors, policy level officials from government as well as donor agencies working in the context of pandemic whereas peers included the co-authors involved in the study. A major part of the questionnaire (for assessing the perceived support) was adapted from the Multidimensional Scale of Perceived Social Support (MSPSS) [19]. The instrument, initially developed in English, was translated into the Nepali version. To ensure the meaning and the intent of the initial set of questions developed in English was not altered we then back translated the questionnaire to English. The survey form was circulated through email and social media like Facebook messenger, skype and viber. Emails of the participants within the network was used. Informed consent was taken from the participants before proceeding with the survey. A consent form detailing the objectives, methodology, and the rights of the participants to decline was attached with the questionnaire. The system was designed in such a way that if the participants did not consent for the survey, there was an option to submit the form without going to the section containing the main survey questions after completing the consent part. If the participant agreed to participate, the system allowed the participants to fill the questions and complete the survey. In addition, they had the right to skip any question if they did not feel like answering the question, however, this would still allow them to answer rest of the questions in the survey form. All the completed survey forms were reviewed daily for completeness and were checked for correctness and accuracy by the researcher. The response was made unavailable after obtaining the required number of participants.

## Measurements

### Perceived social support

To assess the perceived social support, we used the MSPSS as explained above. The MSPSS is a 12-item scale that assesses perceived support from three sources family, friends, and a significant other (e.g., spouse or best friend) [13] using 7-point Likert scale, ranging from 1 as very strongly disagree to 7 as very strongly agree. The MSPSS assesses both perceived availability and adequacy of emotional and instrumental support. This instrument is brief, easy to administer, and has been found to be reliable and valid in various populations and languages. The reliability and validity of the MSPSS have been demonstrated in few other previous studies as well [19–23].

### Compliance with stay-at-home order

Personal compliance with self-isolation was assessed using a single screening question [24]. Linked to the degree to which the participants were isolating themselves from non-household members: “during the past two weeks, to what extent did you limit your in-person contact with people outside your household?” This item was scored on a 5-point scale, ranging from 1 “not at all” to 5 “a great deal” which was further dichotomized. The responses from 1 to 3 were coded as “1” and 4 and 5 as “0” where 0 represents good compliance and 1 represents poor compliance. Further, to assess the level of compliance participants were asked “how many times did you leave home for each of the following purposes during the last week? Possible responses were graded on a 3 point scale with options of “never or once, two or three times, and more than three times” [13, 25].

### Data management and analysis

All completed online survey forms were reviewed routinely for completeness, correctness, and accuracy by the researcher. Data compilation, checking, editing, and coding were carried out following completion of data collection. The responses in the excel sheet were extracted and subsequently exported to SPSS version 20 for further analysis. Descriptive statistics (frequency, mean and standard deviation) were presented in the frequency table. Inferential statistics such as the chi-square test were applied to test the significance of the association between independent and dependent variables. Bivariate analysis was used to show the association between the dependent (perceived support and compliance with stay-at-home order) and independent variables (age, sex, occupation, education, COVID-19 status, COVID-19 vaccination). The significance level was set at 0.05 for Pearson chi-square test.

### Ethical consideration

The study was conducted after obtaining approval from the Ethical Review Board (ERB) of the Nepal Health Research Council. Informed consent was obtained from each participant prior to data collection as a part of the online survey. The information given by the participants was kept confidential by giving a code number instead of the participant’s name while entering data and the description of results in the manuscript didn’t reveal any findings regarding the identity of the participants. Also, the participant’s dignity was maintained by being given the right to reject or discontinue the research study at any time.

## Results

In total, 250 participants were approached to participate in the study through email (50) and social media (200). Out of that, 45 responded to the emails and 174 responded through social media with the shared link making a total of 219 responses. Out of 219 responses, only 214 consented for participation in the study yielding a response rate of 85.6%.

Descriptive characteristics of the participants are depicted in Table 1. The age of the participants ranged from 18 to 62 years (Mean ± SD: 29.4 ± 7.3). More than half (52.8%) of them were male. Nearly half (47.4%) of the participants were skilled/technical workers followed by more than one-third (37.4%) being housewives/students/unemployed. More than four-fifths (82.7%) of the participants had completed bachelors and higher-level education. During the survey, more than half (55.1%) reported their COVID-19 status to be negative while more than one-fourth (28.0%) were unknown about their status and 16% reported being either positive or previously tested positive. Also, still about half (49.1%) of the participants were yet to be vaccinated. The overall mean score of perceived support was found 5.6 ± 1.0. The mean score was higher (Mean ± SD: 6.1 ± 1.1) for support from family and lower (Mean ± SD: 5.2 ± 1.3) for support from friends. Overall, two-fifths of participants (40.2%) reported poor compliance with stay-at-home order.

**Table 1:** Descriptive characteristics of the participants

Characteristics	Frequency (n)	Percent (%)
**Age Mean ± SD**	29.4 ± 7.3	
**Sex**		
Male	113	52.8
Female	101	47.2
**Occupation**		
Business/Self-employed/Service	32	15.0
Housewife/Student/Unemployed	80	37.4
Skilled/Technical labor	102	47.7
**Education**		
Up to 12th grade	37	17.3
Bachelor and above	177	82.7
**COVID-19 status**		
Positive and previously positive	36	16.8
Negative	118	55.1
Don’t know	60	28.0
**COVID-19 vaccination**		
Yes	109	50.9
No	105	49.1
**Perceived social support**	**Mean ± SD**	
Total	5.6 ± 1.0	
Family	6.1 ± 1.1	
Friends	5.2 ± 1.3	
Significant other	5.4 ± 1.4	
Compliance (n = 210)*		
Good compliance	124	57.9
Poor compliance	86	40.2

*Don’t know = 4

Bivariate analyses of socio-demographic characteristics and COVID-19 status with perceived support and compliance with stay-at-home order are presented in Tables 2 and 3, respectively. Further to that, the analysis of association between perceived support and compliance with stay-at-home order is presented in Table 4. A significant difference was observed between sex and perceived support (p value < 0.05) with female participants reporting higher (80.8%) perceived support from family, friends, and significant others in comparison to male participants. Similarly, compliance with stay-at-home order was higher (67.6%) among participants who were not vaccinated against COVID-19 compared to those who were vaccinated and the difference was statistically significant (p value < 0.05). However, no significant differences were observed between other characteristics and perceived support and compliance with stay-at-home order. This study reported that the participants with low perceived support reported higher compliance with stay-at-home order; family (66.7%), friends (85.7%), and significant others (60.0%) although the finding was not significant.

**Table 2:** Association of socio-demographic characteristics and COVID-19 status with perceived support

Characteristics		Perceived support		p value
Sex		High n (%)	Moderate and low n (%)	
	Male	74 (66.7)	37 (33.3)	0.021*
	Female	80 (80.8)	19 (19.2)	
**Occupation**				
	Business/Self-employed/Service	24 (75.0)	8 (25.0)	0.777
	Housewife/Student/Unemployed	55 (70.5)	23 (29.5)	
	Skilled/Technical labor	75 (75.0)	25 (25.0)	
**Education**				
	Up to 12th grade	24 (64.9)	13 (35.1)	0.199
	Bachelor and above	130 (75.1)	43 (24.9)	
**COVID-19 status**				
	Positive and previously positive	29 (82.9)	6 (17.1)	0.360
	Negative	83 (72.2)	32 (27.8)	
	Don’t know	42 (70.0)	18 (30.0)	
**COVID-19 vaccination**				
	Yes	80 (74.1)	28 (25.9)	0.803
	No	74 (72.5)	28 (27.5)	

*significant at p < 0.05, Pearson chi-square

**Table 3:** Association of socio-demographic characteristics and COVID-19 status with compliance with stay-at-home order

Characteristics		Compliance		p value
Sex		Good n (%)	Poor n (%)	
	Male	71 (64.5)	39 (35.5)	0.089
	Female	53 (53.0)	47 (47.0)	
**Occupation**				
	Business/Self-employed/Service	15 (46.9)	17 (53.1)	0.272
	Housewife/Student/Unemployed	46 (59.0)	32 (41.0)	
	Skilled/Technical labor	63 (63.0)	37 (37.0)	
**Education**				
	Up to 12th grade	21 (58.3)	15 (41.7)	0.924
	Bachelor and above	103 (59.2)	71 (40.8)	
**COVID-19 status**				
	Positive and previously positive	24 (66.7)	12 (33.3)	0.529
	Negative	67 (58.8)	47 (41.2)	
	Don’t know	33 (55.0)	27 (45.0)	
**COVID-19 vaccination**				
	Yes	55 (50.9)	33 (49.1)	0.014*
	No	69 (67.6)	33 (32.4)	

*significant at p < 0.05, Pearson chi-square

**Table 4:** Association of perceived support and compliance with stay-at-home order

Characteristics		Compliance		p value
Perceived support		Good n (%)	Poor n (%)	
Family	High	107 (59.8)	72 (40.2)	0.803
	Moderate	15 (53.6)	13 (46.4)	
	Low	2 (66.7)	1 (33.3)	
Friends	High	72 (60.5)	47 (39.5)	0.051
	Moderate	40 (51.9)	37 (48.1)	
	Low	12 (85.7)	2 (14.3)	
Significant other	High	84 (60.4)	55 (39.6)	0.787
	Moderate	28 (54.9)	23 (45.1)	
	Low	12 (60.0)	8 (40.0)	

## Discussion

The present study aimed to assess the perceived social support and compliance with stay-at-home order during the COVID-19 pandemic in Nepal.

Using the MSPSS, the results of this study found that overall perceived support was found to be higher for a family in comparison to friends and significant other. This may be due to existing socio-cultural practices in the Nepalese society whereby family has continuous involvement and care in every part of individual’s life which might have led to the participant’s perception of better support from family than other groups. Supporting the findings from our study, a study conducted among Nepalese adolescents revealed their orientation towards support from family members than others [26].

Family may play a key role in shaping personal beliefs, attitudes and behavior in relation to health. Evidence from literature around family support and social behavior indicates that the family is depicted as a supportive network playing a significant role in adopting healthy behaviors [16]. Family members may serve as a source of support in adopting preventative measures during emergencies by promoting health behavior [27]. Social support from family is found to have positive influence on life satisfaction of the individuals [28] and is found to promote healthy behavior through social control, support and other interventions [29].

In our study, the majority of the participants were found practicing social distancing measures during COVID-19 pandemic. In order to prevent and slow the transmission of COVID-19, the Government of Nepal implemented SMS (social distancing, mask, and sanitization) measures. Similar findings of a higher level of compliance (70%) with government-imposed public health measures were reported in a study in South Africa [30] and Spain [31]. However, a contrasting finding was reported by a study conducted in the Kathmandu valley, Nepal which concluded people did not follow enough social distancing measures in public places, hospitals, and vehicles, calling for consistent monitoring in public places and strict implementation of the measures at the institutional level [32]. While deterrence played little role, fear of transmission to family members, concern for self-health and family, desire to maintain a normal life and fear of economic loss might [15, 33], perceived risk of the stigma associated with COVID-19, trust in the government’s ability to deal with disease prevention [34] may have led to higher compliance with stay-at-home order in our study.

In regard to overall perceived support from family, friends, and significant others, female participants reported higher perceived support than males in our study. Supporting this finding, a study conducted among college students in Columbia showed female participants were more likely than their male counterparts to report high perceived social support (75% vs. 50%, *p* = 0.002) [35]. Higher perceived support among female participants may be due to the fact that women tend to have larger social networks than men and are likely to receive support from different sources while men are solely dependent upon their partners [36].

Regarding compliance with stay at home, significant differences were observed in vaccinated and unvaccinated individuals. Compliance was found higher among participants who were not vaccinated against COVID-19 in contrast to the already vaccinated participants. Though there is no supporting evidence of reduced compliance among individuals after COVID-19 vaccination [37]; higher compliance among unvaccinated individuals in our study may be mainly due to fear of COVID-19 transmission.

Our main variable of interest was perceived social support. Although there is plenty of literature suggesting positive influence of social support on adoption of healthy behavior, we interestingly didn’t find any association between perceived support and compliance in our study. One reason behind this maybe the participants of the study were possibly not satisfied with the support they have received. Their perception on the type of support received might have also determined their intention to reflect into behavior and possibly affecting the compliance. They might have wanted emotional support but received instrumental or vice-versa. Besides, gender, ethnicity and cultural factors may greatly influence their perception of social support [36, 37]. Though, we did not find a significant association of perceived support and compliance, this might still be important in terms of public awareness messaging based on the literatures available. It might however be needed to be contextualized to the local context and cultural settings.

This study provides insights into factors affecting compliance with stay-at-home order during COVID-19 pandemic. However, some limitations of the study should be considered, as the COVID-19 pandemic issued a lockdown worldwide, this limited the possibility of following appropriate techniques for sampling as well as data collection. Only people who have access to the internet and are friendly in using it were included which limits the generalization of these findings to the public of Nepal and other countries. Furthermore, self-report information collected from participants is subjected to recall and social-desirability biases.

## Conclusion

Overall, the results of this study suggest that perceived support from family is higher compared to others. More evidence might be needed to establish what factors affect the compliance with public health measures. Contextual public awareness messaging could still be important considering the contextual factors. This evidence might prove worthy when considering a comparison amongst assessment results of diverse cultures, origins and in formulating policy during emergencies of similar nature as well. However, more in-depth studies on the subject matter possibly employing qualitative methods as well would be fruitful in understanding the impact of such mandatory orders on comprehensive human health.

## Electronic supplementary material

Below is the link to the electronic supplementary material.

Supplementary Material 1

## Data Availability

Dataset of this study is provided in this article as a supplementary file.

## List of abbreviations

SARS-CoV-2: Severe Acute Respiratory Syndrome Coronavirus-2
COVID-19: Coronavirus Disease-2019
WHO: World Health Organization
MSPSS: Multidimensional Scale of Perceived Social Support
SPSS: Statistical Package for Social Sciences
SD: Standard Deviation
ERB: Ethical Review Board
